# Widespread closure of HIV prevention and care services places youth at higher risk during the COVID-19 pandemic

**DOI:** 10.1371/journal.pone.0249740

**Published:** 2021-09-10

**Authors:** Rob Stephenson, Alison R. Walsh, Tanaka M. D. Chavanduka, Gregory Sallabank, Keith J. Horvath, Amanda D. Castel, Erin E. Bonar, Lisa Hightow-Weidman, Jose A. Bauermeister, Patrick S. Sullivan

**Affiliations:** 1 Department of Systems, Populations and Leadership, School of Nursing, University of Michigan, Ann Arbor, MI, United States of America; 2 Center for Sexuality and Health Disparities, University of Michigan, Ann Arbor, MI, United States of America; 3 Department of Psychology, San Diego State University, San Diego, CA, United States of America; 4 Department of Epidemiology, The George Washington University Milken Institute School of Public Health, Washington, DC, United States of America; 5 Addiction Center, Department of Psychiatry, University of Michigan, Ann Arbor, MI, United States of America; 6 Injury Prevention Center, University of Michigan, Ann Arbor, MI, United States of America; 7 Institute for Global Health and Infectious Diseases, University of North Carolina at Chapel Hill, Chapel Hill, NC, United States of America; 8 Department of Family and Community Health, School of Nursing, University of Pennsylvania, Philadelphia, PA, United States of America; 9 Department of Epidemiology, Rollins School of Public Health, Emory University, Atlanta, GA, United States of America; Beth Israel Deaconess Medical Center/Harvard Medical School, UNITED STATES

## Abstract

**Background:**

Central to measuring the impact of the COVID-19 pandemic on HIV is understanding the role of loss of access to essential HIV prevention and care services created by clinic and community-based organization closures. In this paper, we use a comprehensive list of HIV prevention services in four corridors of the US heavily impacted by HIV, developed as part of a large RCT, to illustrate the potential impact of service closure on LGBTQ+ youth.

**Methods:**

We identified and mapped LGBTQ+ friendly services offering at least one of the following HIV-related services: HIV testing; STI testing; PrEP/PEP; HIV treatment and care; and other HIV-related services in 109 counties across four major interstate corridors heavily affected by HIV US Census regions: Pacific (San Francisco, CA to San Diego, CA); South-Atlantic (Washington, DC to Atlanta, GA); East-North-Central (Chicago, IL to Detroit, MI); and East-South-Central (Memphis, TN to New Orleans, LA).

**Results:**

There were a total of 831 LGBTQ+ youth-friendly HIV service providers across the 109 counties. There was a range of LGBTQ+ youth-friendly HIV-service provider availability across counties (range: 0–14.33 per 10,000 youth aged 13–24 (IQR: 2.13), median: 1.09); 9 (8.26%) analyzed counties did not have any LGBTQ+ youth-friendly HIV service providers. The Pearson correlation coefficient for the correlation between county HIV prevalence and LGBTQ+ youth-friendly HIV service provider density was 0.16 (p = 0.09), suggesting only a small, non-statistically significant linear relationship between a county’s available LGBTQ+ youth-friendly HIV service providers and their HIV burden.

**Conclusions:**

As the COVID-19 pandemic continues, we must find novel, affordable ways to continue to provide sexual health, mental health and other support services to LGBTQ+ youth.

## Introduction

The COVID-19 pandemic was caused by the 2019 novel coronavirus (SAR-CoV-2) which has experienced rapid global spread since it was first identified in China in late 2019 [1, 2]. Since the first COVID-19 cases were identified in the United States (U.S) in March 2020, COVID-19 has spread to all 50 states, and, as of March 2021, there were 28, 456, 860 confirmed cases resulting in 513, 122 deaths [1]. The distribution of COVID-19 cases is non-uniform throughout the U.S, with 13 states recording more than 40,000 cases (California, Texas, Louisiana, Georgia, Florida, Illinois, Michigan, Virginia, Pennsylvania, Maryland, New York, Massachusetts, and Connecticut) [1]. Recent evidence has illustrated the emergence of strong disparities within the COVID-19 pandemic in the U.S: COVID-19 has disproportionately affected communities of color [3–5], driven by lack of access to testing and economic conditions that limit opportunities for social distancing. The geographic and racial disparities observed in the US COVID-19 pandemic are similar to those observed in the U.S HIV pandemic, which is similarly geographically non-uniform (concentrated in large urban areas and the Southern states), with significantly higher incidence among communities of color [6].

The similarity in HIV and COVID-19 epidemiology in the U.S has led to several recent reports calling for urgent investigations into the synergies between the two pandemics [7–14]. Mandated stay-at-home orders and the resultant closure of many clinics and community-based organizations have significantly reduced access to routine HIV testing and other HIV prevention options such as condoms or pre-exposure prophylaxis (PrEP) [11, 15]. Although there is some evidence that advances in telehealth have to some extent replaced in-person HIV prevention and care services [16, 17], access to telehealth services are non-uniform and dependent on patient’s access to technology. While self-testing for HIV using home-testing kits is available, reduced or lost employment and the resultant income reduction and issues surrounding the safe delivery of testing kits may limit home-testing as a viable option for many individuals. People living with HIV may be more likely to contract COVID-19 at younger ages, possibly due to a higher prevalence of respiratory and cardiovascular co-morbidities [18–20]. The closure of clinics and community-based organizations, unemployment, and financial stress have the potential to significantly impact both HIV and COVID-19 outcomes by limiting access to routine HIV care and anti-retroviral therapy (ART) [11, 15, 21]; data have demonstrated the impacts of such closures amongst gay and bisexual men who have sex with men (GBMSM) [22]. People living with HIV also have reported high levels of anxiety related to perceived COVID-19 risk and increased social isolation [23, 24]. Not only does this increase the need for mental health services in this population, it could potentially further reduce access and adherence to care, if individuals avoid healthcare facilities and providers out of caution.

Although opportunities for HIV prevention and care may be reduced by the need for physical distancing to curb the spread of COVID-19, there is evidence that individuals continue to engage in HIV risk behaviors. In a sample of 105I U.S men who have sex with men (MSM) (conducted from April 2–13, 2020), Sanchez et al. [22] found that while 51% reported a decrease in the number of sex partners during the COVID-19 pandemic, 48% reported no change in their number of sex partners. The use of apps/websites to find sex partners remained high, with 49% reporting no change in the use of these sites. Participants almost universally reported that they had no change in access to (98%) or use of (92%) condoms. However, 10% of participants reported an increase in their use of non-prescription drugs. Carrico et al. [7] note that the co-occurrence of methamphetamine use and HIV among MSM could create a double jeopardy for COVID-19, amplifying biological and behavioral risk for infection with COVID-19. Liu et al. [25] note that U.S youth are experiencing high levels of loneliness, COVID-19-specific worry, and low distress tolerance, with 43.3% reporting depressive symptomology reflective of likely major depressive disorder (PHQ-8 scores ≥ 10). These high levels of poor mental health among youth have the potential to translate into negative coping behaviors, such as substance misuse [26, 27], increasing the risk for the acquisition of HIV.

Central to understanding the impact of the COVID-19 pandemic on HIV in the U.S is understanding the role of loss of access to essential HIV prevention and care services created by clinic and community-based organization closures. In this paper, we use data from a large, randomized controlled trial for adolescent GBMSM aged 13–18 years to map HIV prevention services in four corridors of the U.S heavily impacted by HIV [28]. By mapping HIV prevalence and service availability data for young GBMSM, this paper aims to take a first step in illustrating areas that, prior to the COVID-19 epidemic, had limited service availability and thus are likely to experience significant disruptions in HIV prevention and care services during the COVID-19 pandemic.

## Methods

iREACH is a randomized control trial (RCT) (Clinical Trials Registration number NCT03155841) that aims to test the efficacy of an e-delivered life skills intervention on cognitive and behavioral HIV-related outcomes for adolescent GBMSM (ages 13–18), in 109 counties across four major interstate corridors heavily affected by HIV (U.S Census regions: Pacific (San Francisco, CA to San Diego, CA; 14 counties); South-Atlantic (Washington, DC to Atlanta, GA; 57 counties); East-North-Central (Chicago, IL to Detroit, MI; 11 counties); and East-South-Central (Memphis, TN to New Orleans, LA; 27 counties)) [28]. The iREACH intervention is a web-app that includes a searchable database of LGBTQ+ youth-friendly public and private health and well-being service providers and resources located within the four iREACH study corridors. Resources were included in this database if they: 1) were located in one of the 109 counties within the study’s recruitment corridors; 2) report that they provided youth-friendly health or well-being services or services that were specifically developed and targeted for youth; and 3) indicated on their website that they were accepting/ friendly of sexual or gender minority clients. Within the database, providers are identified according to their specific service offering(s) from among the following (each organization may provide more than one service): a) HIV testing, STI testing, and PrEP/PEP; b) HIV treatment and care; c) mental health services; d) intimate partner violence (IPV) support; e) support group(s); f) substance use/abuse services; g) LGBTQ+-directed programming; h) food pantry; i) housing/shelter; j) school resource(s); and k) family support services. Providers’ location and available service(s) are verified on a quarterly basis by study staff via email, web, and/or telephone calls to service agencies.

For the current analysis, we identified providers within this database offering at least one of the following HIV-related services: HIV testing, STI testing, or PrEP/PEP and HIV treatment and care. Using this list of LGBTQ+ youth-friendly HIV-service providers, we counted the number of unique services in each of the 109 analyzed counties to assess the availability of HIV services. A container-based approach was used to create a proxy measure of county-level HIV service availability; each county’s *per capita HIV service provider density* was calculated by dividing the provider count by the county’s 2018 youth population (aged 13–24). County-level population data was obtained from the American Community Survey 2013–2017: 5-year population estimates [29] and denominator totals were for ages 13–24 years, calculated by adding 2/5^ths^ of the 10–14 age grouping to the 15–19 and 20–24 age groupings.

County-level HIV prevalence data (for those ages 13 and older) was obtained from AIDSVu’s 2018 HIV prevalence estimates [30, 31]; detailed descriptions of these data methods are available on the AIDSVu website [32]. Data for ages 13 and older were used rather than data for the 13–24 year age group as 89 out of the 109 counties had significant levels of missing data for HIV prevalence for the 13–24 year old group. For counties with AIDSVu-suppressed HIV case count and prevalence data (counties with between 1 and 4 prevalent HIV cases in persons aged 13 or older; N = 34 (31%)), the HIV case count was set to 2.5 and prevalence was calculated per 100,000 population aged 13 and up; the population was calculated by subtracting the population of children under 13 from the county’s total population. Although county HIV prevalence rates are not independent observations, we assessed the Pearson correlation coefficients and associated p-values to estimate the correlation between county HIV prevalence and LGBTQ+ youth-friendly HIV service provider density across the 109 analyzed counties.

Descriptive analyses were conducted in SAS 9.4 (Cary, NC). HIV service provider availability, or density, and county-level HIV prevalence (ages 13 and up) was mapped using Kepler GL, an open source geo-analytic software [33]. County level attributes were matched geometrically to the 2017 US Census shape-file using the five-digit Federal Information Processing Standard county code.

The research and ethics presented in this study were approved by the IRB of the University of North Carolina at Chapel Hill (16–3183).

## Results

**Fig 1:**
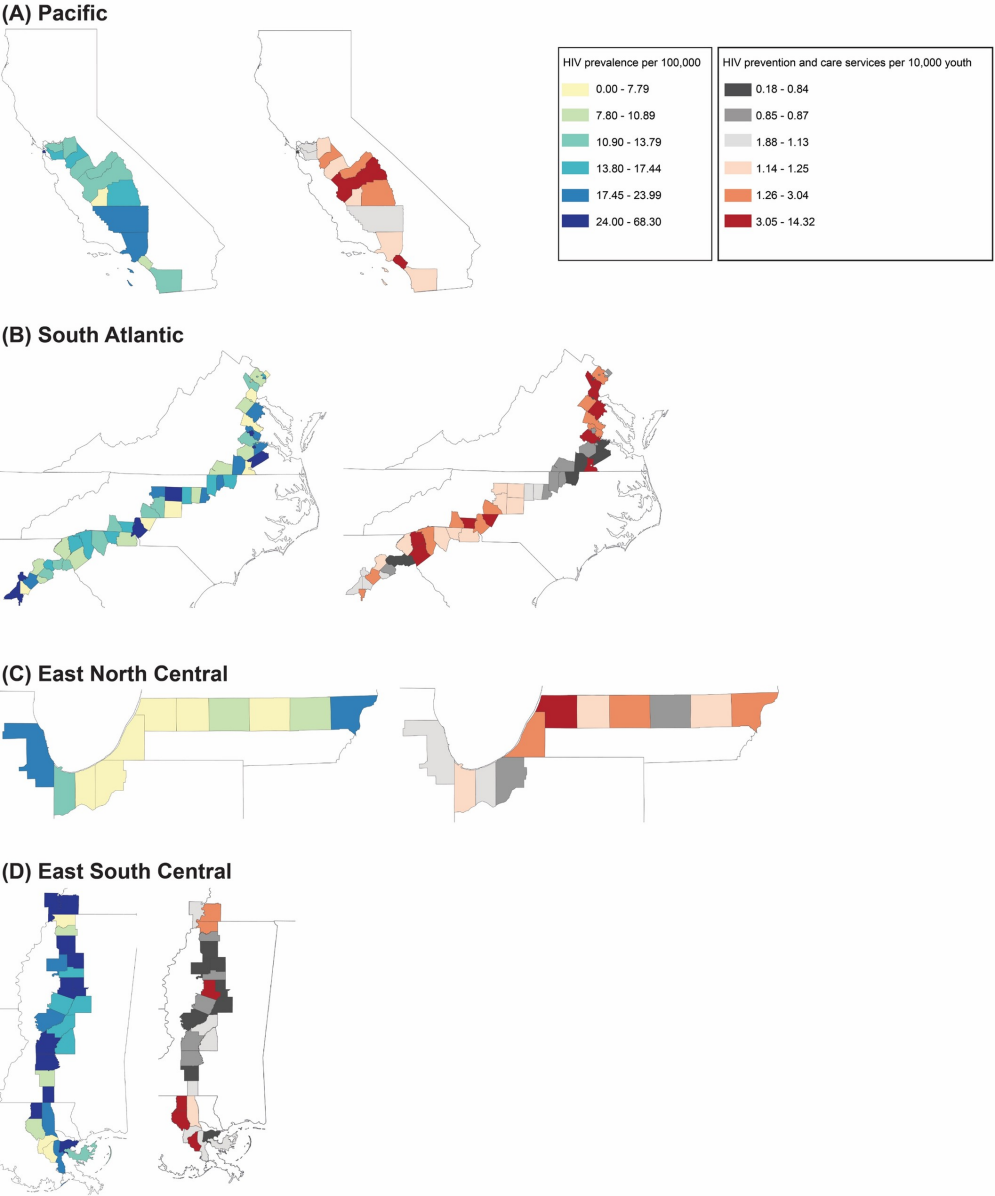
Mapping of county level HIV prevalence and HIV service providers densities in A Pacific (San Francisco, CA to San Diego, CA) B South-Atlantic (Washington, DC to Atlanta, GA;) C East-North-Central (Chicago, IL to Detroit, MI) and D East-South-Central (Memphis, TN to New Orleans, LA). County HIV prevalence and HIV service provider density.

There were a total of 831 LGBTQ+ youth-friendly HIV services offering HIV testing, STI testing, PrEP/PEP, HIV treatment/care, and/or other HIV services across the 109 analyzed counties. Fig 1 shows choropleth maps of HIV prevalence and the spatial distribution of HIV service provider density per 10,000 youth in each study area, demonstrating a concentration of counties with high HIV prevalence and low HIV services for youth, particularly in the South-Atlantic and South-East-Central regions. Across all analyzed counties, HIV prevalence for persons aged 13 or older ranged from 0 to 68.30 cases per 100,000 population (aged 13 or older; median: 13.80; IQR: 11; mean (standard deviation (SD)): 16.65 (11.78)). In the Pacific region, county prevalence ranged from 7.4 (Kings County, CA) to 29.9 (San Francisco County, CA) cases per 100,000 (median: 13.55 cases/100,000). In the South-Atlantic region county prevalence ranged from 0 to 68.3 (Fulton County, GA) cases per 100,000 (median: 13.70 cases/100,000). East-North-Central counties had HIV prevalence between 1.92 (Berrien County, MI) and 22.2 (Cook County, IL) cases per 100,000 (median: 6.70 cases/100,000). The maps show that counties in the East-South-Central region had higher HIV rates for this age group than other regions. East-South-Central counties had HIV prevalence between 1.77 (DeSoto County, MS) and 53.7 (Orleans Parish, LA) cases per 100,000 (median: 20.10 cases/100,000). Fulton and Clayton Counties in Georgia had the highest HIV prevalence of all 109 counties (68.30 and 59.30 cases per 100,000 population 13 and older, respectively).

There was a range of LGBTQ+ youth-friendly HIV-service provider availability across counties (range: 0–14.33 per 10,000 youth aged 13–24 (IQR: 2.13), median: 1.09); 9 (8.26%) analyzed counties did not have any LGBTQ+ youth-friendly HIV service providers—4 in Virginia, 3 in Louisiana, 1 in Michigan, and 1 in Mississippi. In the Pacific region, county HIV provider density ranged from 0.33 (Fresno County, CA) to 4.82 (San Francisco County, CA) per 10,000 youth (median: 0.92 providers/10,000). In the South-Atlantic region, county HIV provider density ranged from 0 (Caroline, Greensville, Colonial Heights City, and Hopewell City Counties, VA) to 14.33 (Sussex County, VA) per 10,000 youth (median: 1.03 providers/10,000). East-North-Central county HIV provider density ranged from 0 (Van Buren County, MI) to 2.80 (Jackson County, MI) per 10,000 youth (median: 1.02 providers/10,000). East-South-Central HIV provider density ranged from 0 (Livingston Parish, St. Charles Parish, St. Helena Parish, LA, Carroll County, MS) to 13.52 (Montgomery County, CA) per 10,000 youth (median: 1.78 providers/10,000).

The Pearson correlation coefficient for the correlation between county HIV prevalence and LGBTQ+ youth-friendly HIV service provider density was 0.16 (*p* = 0.09), suggesting only a small, non-statistically significant linear relationship between a county’s available LGBTQ+ youth-friendly HIV service providers and their known HIV burden. Aside from Greensville County, VA, which had no known HIV cases in 2018, the counties without any LGBTQ+ youth-friendly HIV service providers had HIV prevalence between 3.99 and 28.53 cases/100,000 and Caroline County, VA, St. Charles and St. Helena Parishes, LA, and Carroll County, MS all had HIV prevalence >15 cases/100,000. Among the 100 counties that had at least one LGBTQ+ youth-friendly HIV provider, 4 had fewer than 2.5 LGBTQ+ youth-friendly HIV service providers per 10,000 youth and HIV prevalence greater than 40 cases per 100,000 persons aged 13 or older [Petersburg City, VA; Hinds County (MS); Clayton County (GA); Orleans Parish (LA)].

## Discussion

The results demonstrate that prior to the onset of the COVID-19 pandemic there were generally low levels of availability of LGBTQ+ friendly HIV prevention and care services for youth in four corridors of the U.S that are known to be heavily impacted by HIV. Although the distribution of HIV prevalence for those 13 years of age and older reflects what is already known about the geographic distribution of HIV in the U.S [34], the mapping of LGBTQ+ youth friendly services demonstrates that service provision is not keeping pace with demand, and this lack of service availability it likely heightened during the pandemic. A small, but not insignificant number of counties (8%) had no services available: in these counties GBMSM youth must either travel long distances to receive services (limiting access among those without the resources for travel) or must rely on online sources of information.

While several previous studies have noted the issues LGBTQ+ youth face in accessing HIV prevention and care services (i.e., concerns around cost or privacy [35, 36]), this current paper demonstrates that prior to the COVID-19 pandemic many youth experienced a fundamental lack of age- and culturally-appropriate services. There was a weak, but insignificant, association between the presence of services and HIV prevalence, illustrating that services are not consistently targeted towards areas of highest demand. Areas with the highest prevalence of HIV are often those that experience high degrees of other structural vulnerability indicators (i.e., high rates of poverty and unemployment), reinforcing the need to provide easily accessible LGBTQ+ friendly HIV prevention and care services for youth in these areas.

In terms of the current COVID-19 pandemic, the closure of service providers in these regions has likely negatively impacted already sparse existing resources for LGBTQ+ youth. Although some regions are slowly reopening their services, the loss of employment or other resources during stay-at-home orders may mean that these services are no longer available or desirable to youth. There is clearly a need to make culturally and age relevant, economically accessible, and geographically proximate HIV prevention and care services available to youth and to ensure youth are aware that these services exist and how they can access them. In addition to creating new services in areas in which they currently do not exist, the focus should be on finding new modes of service delivery for youth in areas of high vulnerability. Hightow-Weidman et al. [37] recently noted the potential for telehealth interventions to surmount many of the barriers to service delivery created by the COVID-19 pandemic, and it may be possible that many HIV service providers have already moved towards telehealth delivery, like other specialties [38–40]. Several recent studies have successfully implemented telehealth interventions to provide HIV prevention and care services to youth and adults living in areas with low service availability [41, 42]. Online-delivered interventions offer user flexibility and efficiency, and combined with the collection of home bio-marker specimens (e.g., for STI testing or viral load) can offer the same breadth and quality of services as in-person delivery. This service format should also include ensuring youth have adequate supplies of PrEP or ART delivered through the use of mail order pharmacies or other mechanisms that don’t require in-person clinic attendance.

There are two key limitations to the current analysis. First, inclusion in the database was dependent on services listing on their websites that they specifically provided services for youth and sexual and gender minority clients. Services not including this information publicly would not be included in the database. Second, due to high degrees of missing data on HIV prevalence for the 13–24 age group in many counties, service availability was compared to the HIV prevalence for ages 13 and older, limiting a direct comparison of HIV prevalence and service availability for youth aged 13–24 years.

As the COVID-19 pandemic continues, we must find novel, affordable ways to continue to provide sexual health, mental health and other support services to LGBTQ+ youth. Youth continue to engage in risk behaviors, and heightened negative mental health contexts created by the pandemic may precipitate risk-taking as a coping response. The results presented here demonstrate areas of high need across the U.S, and provide a call to action for service providers, donors and policymakers, to think critically about the importance of providing services to youth in these areas. Providing culturally appropriate services to youth—through both telehealth and in-person modes–is foundational to the success of the Ending the HIV Epidemic goals [43].
